# Supplementary material for: N-SREBP2 Provides a Mechanism for Dynamic Control of Cellular Cholesterol Homeostasis
Source: Cells. 2024 Jul 25;13(15):1255. doi: 10.3390/cells13151255 (PMC11311687; doi:10.3390/cells13151255)
Supplement: Supplementary file 1 [file cells-13-01255-s001.zip › cells-3082814-supplementary.pdf]

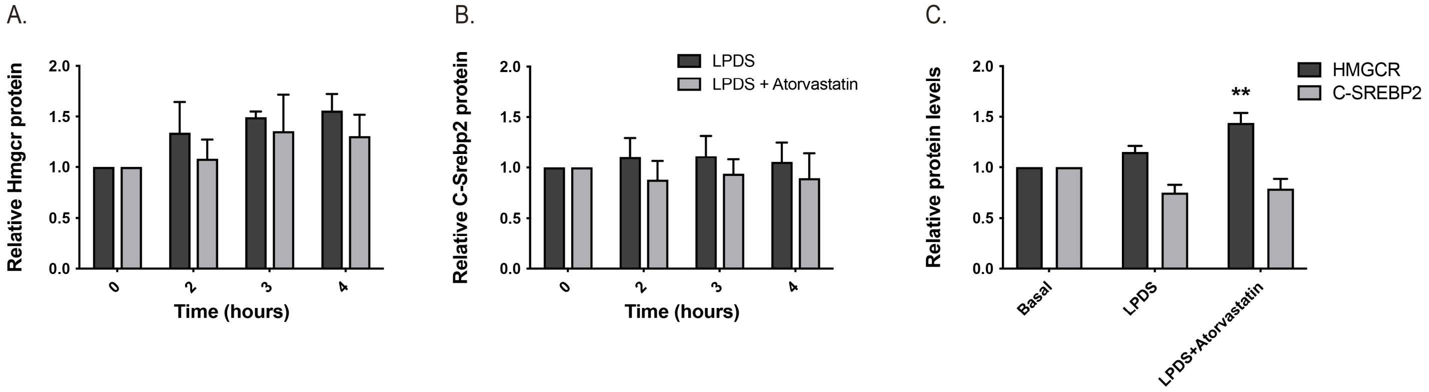

Figure S1. Atorvastatin does not produce a larger effect on HMGCR protein levels in McArdle-RH7777 cells switched to lipoprotein-depleted media for only 4-h. Western blot analysis of HMGCR (A) and C-SREBP2 (B) in whole-cell lysates of McArdle-RH7777 cells switched from basal media (0-h) to media containing lipoprotein-depleted serum (LPDS) and LPDS plus 1mM atorvastatin for the indicated time points. Three independent experiments, each containing three technical repeats were performed. Data (mean ± SEM) normalised to b-actin are expressed relative to basal values. (C). Equivalent analyses for McArdle-RH7777 cells switched from basal media (0-h) to indicated media for 24-h. Values are from four independent experiments, each containing three technical repeats. Statistical differences from 0-h were determined by one-way ANOVA followed by Dunnet's multiple comparison test. Significance is indicated by asterisks: ** $p < 0.01$.

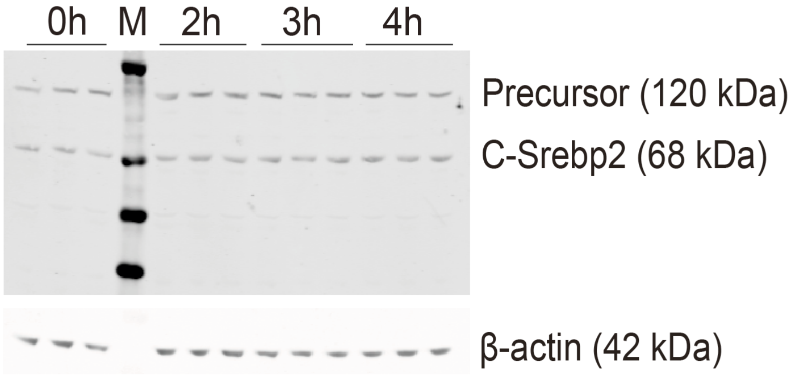

Figure S2. Immunoblot of C-SREBP2-containing sequences in McArdle-RH7777 cells. Representative Western Blot of precursor SREBP2 and of the C-SREBP2 fragment in three individual sets of whole cell lysates of McArdle-RH7777 cells switched from basal media (0-h, lanes 1-3) to media containing lipoprotein-depleted serum (LPDS) for the indicated time points (lanes 5- 13). M = size marker.

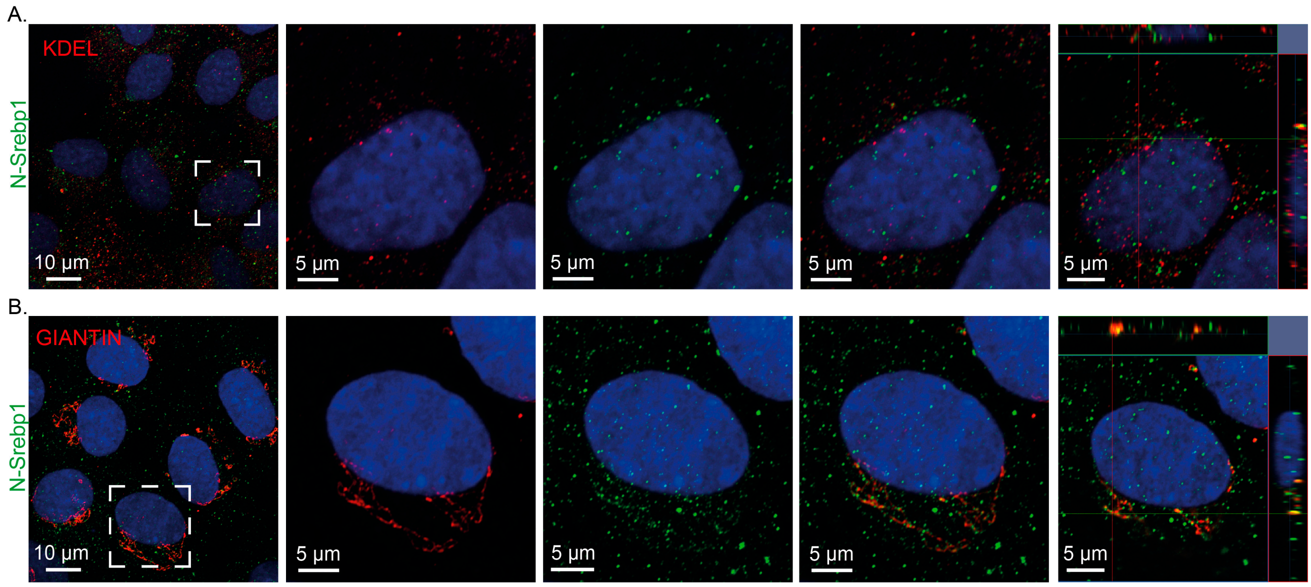

Figure S3. Localization of Endogenous N-SREBP1 in McArdle-RH7777 cells. Cells cultured under basal conditions were subject to indirect immunofluorescence using specified antibodies and counterstained with DAPI. Left-hand panels, maximum intensity projection images of representative field of cells. Right-hand panels, show expanded view of region highlighted by white squares. Enlargements show the separate channels (middle panels), and orthogonal views (XZ axis, right-hand side; YZ, top) of merged images through the centre of the nucleus. (**A, B**) N-SREBP1 (green), ER-marker KDEL (red); Golgi- marker Giantin (red).

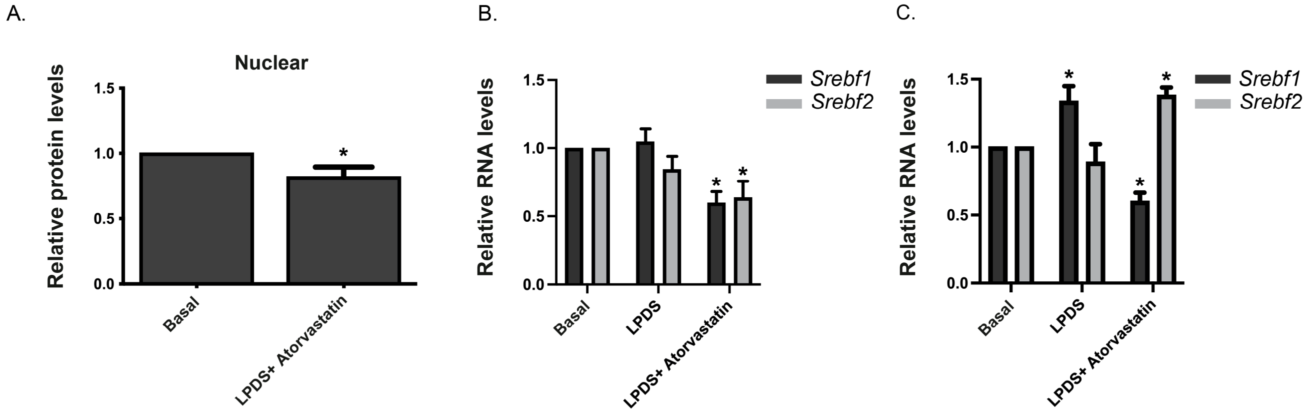

Figure S4. ***Srebf1*****/2 RNA and Nuclear N-SREBP2 Protein Levels in Cells Incubated with Atorvastatin.** (**A**). Quantification of N-SREBP2 protein in nuclear extracts prepared from McArdle-RH7777 cells cultured under basal conditions and cells switched to lipoprotein-depleted media (LPDS) containing 1mM atorvastatin for 10-h. Values represent mean ± standard error, from five independent experiments, each with three replicates. *$p < 0.05$ by Student's t-Test. (**B, C**) RT-qPCR quantification of *Srebf1* and *Srepf2* in total RNA extracted from McArdle-RH7777 cells switched to LPDS and LPDS-atorvastatin-containing media for 11-h (**B**) and 12-h (**C**). Data (mean ± SEM), normalised to *Ppia*, are from three independent experiments, each with three technical replicates. * $p < 0.05$ versus cells at 0-h, determined by one-way ANOVA.

```
SFESGSGGWFDWMMPTLLLWLVNGVIVLSVFVKLLVHGEPVIRPHSRSSVTFWRHRKQAD     582
LDLARGDFAAAAGNLQTCLAVLGRALPTSRLDLACSLSWNVIRYSLQKLRLVRWLLKKVF     642
QCRRATPATEAGFEDEAKTSARDAALAYHRLHQLHITGKLPAGSACSDVHMALCAVNLAE     702
CAEEKIPPSTLVEIHLTAAMGLKTRCGGKLGFLASYFLSRAQSLCGPEHSAVPDSLRWLC     762
HPLGQKFFMERSWSVKSAAKESLYCAQRNPADPIAQVHQAFCKNLLERAIESLVKPQAKK     822
KAGDQEEESCEFSSALEYLKLLHSFVDSVGVMSPPLSRSSVLKSALGPDIICRWWTSAIT     882
VAISWLQGDDAAVRSHFTKVERIPKALEVTESPLVKAIFHACRAMHASLPGKADGQQSSF     942
CHCERASGHLWSSLNVSGATSDPALNHVVQLLTCDLLLSLRTALWQKQASASQAVGETYH     1002
ASGAELAGFQRDLGSLRRLAHSFRPAYRKVFLHEATVRLMAGASPTRTHQLLEHSLRRRT     1062
TQSTKHGEVDAWPGQRERATAILLACRHLPLSFLSSPGQRAVLLAEAARTLEKVGDRRSC     1022
NDCQQMIVKLGGGTAIAAS                                              1141
```

Figure S5. Sites of C-SREBP2 ubiquitination (red). Data extracted from ubiquitinomes of HEK293T cells[71–73].
